# Plaque Bacterial Microbiome Diversity in Children Younger than 30 Months with or without Caries Prior to Eruption of Second Primary Molars

**DOI:** 10.1371/journal.pone.0089269

**Published:** 2014-02-28

**Authors:** He Xu, Wenjing Hao, Qiong Zhou, Wenhong Wang, Zhongkui Xia, Chuan Liu, Xiaochi Chen, Man Qin, Feng Chen

**Affiliations:** 1 Department of Pediatric Dentistry, School of Stomatology, Peking University, Beijing, China; 2 Department of Pediatric Dentistry, Maternal and Child Care Service Centre of Haidian District, Beijing, China; 3 BGI, Shenzhen, Guangdong, China; 4 Center of Bioinformatics and Key Laboratory for Neuroinformation of Ministry of Education, School of Life Science and Technology, University of Electronic Science and Technology of China, Chengdu, Sichuan, China; 5 Department of Oral Microorganism, School of Stomatology, Peking University, Beijing, China; 6 Central Laboratory, School of Stomatology, Peking University, Beijing, China; University of Oklahoma Health Sciences Center, United States of America

## Abstract

**Objective:**

Our primary objective is to phylogenetically characterize the supragingival plaque bacterial microbiome of children prior to eruption of second primary molars by pyrosequencing method for studying etiology of early childhood caries.

**Methods:**

Supragingival plaque samples were collected from 10 caries children and 9 caries-free children. Plaque DNA was extracted, used to generate DNA amplicons of the V1–V3 hypervariable region of the bacterial *16S rRNA* gene, and subjected to 454-pyrosequencing.

**Results:**

On average, over 22,000 sequences per sample were generated. High bacterial diversity was noted in the plaque of children with caries [170 operational taxonomical units (OTU) at 3% divergence] and caries-free children (201 OTU at 3% divergence) with no significant difference. A total of 8 phyla, 15 classes, 21 orders, 30 families, 41 genera and 99 species were represented. In addition, five predominant phyla (*Firmicute, Fusobacteria, Proteobacteria, Bacteroidetes* and *Actinobacteria*) and seven genera (*Leptotrichia, Streptococcus, Actinomyces, Prevotella, Porphyromonas, Neisseria, and Veillonella*) constituted a majority of contents of the total microbiota, independent of the presence or absence of caries. Principal Component Analysis (PCA) presented that caries-related genera included *Streptococcus* and *Veillonella*; while *Leptotrichia*, *Selenomonas*, *Fusobacterium*, *Capnocytophaga* and *Porphyromonas* were more related to the caries-free samples. *Neisseria* and *Prevotella* presented approximately in between. In both groups, the degree of shared organism lineages (as defined by species-level OTUs) among individual supragingival plaque microbiomes was minimal.

**Conclusion:**

Our study represented for the first time using pyrosequencing to elucidate and monitor supragingival plaque bacterial diversity at such young age with second primary molar unerrupted. Distinctions were revealed between caries and caries-free microbiomes in terms of microbial community structure. We observed differences in abundance for several microbial groups between the caries and caries-free host populations, which were consistent with the ecological plaque hypothesis. Our approach and findings could be extended to correlating microbiomic changes after occlusion establishment and caries treatment.

## Introduction

Early childhood dental decay typically affects many teeth, with caries developing rapidly, often soon after eruption. The pattern of caries that affect the primary maxillary incisor and first molar teeth and often spare the mandibular incisor teeth is thought to be related to the eruption times of primary teeth and subsequent acquisition of cariogenic bacteria [1]. In certain groups, children that develop caries before 2.5 years of age usually exhibit decay of the smooth surfaces of maxillary incisors and occlusal fissures of the first molar teeth [2].

Dental caries is a dieto-bacterial disease resulting from interactions among a susceptible host, cariogenic bacteria, and cariogenic diets. The primary pathogens associated with dental caries are *Streptococcus mutans (S. mutans)* and *Streptococcus sobrinus (S. sobrinus),* the *Mutans Streptococci* (MS). Other associated species include non-mutans *Streptococcus*, *Lactobacillus, Actinomyces, Bifidobacterium,* and *Veillonella* species [3]. Studies of early childhood caries microbiota using cultural [4] and molecular approaches [5] [6] have further expanded the range of species detected in caries. *Lactobacillus gasseri*, *Lactobacillus fermentum, Lactobacillus vaginalis*, and *Streptococcus mutans* with *Streptococcus sobrinus* have been demonstrated to be associated with childhood caries.

According to the findings of Caufield *et al.*, the initial acquisition of MS occurs at the median age of 26 months during a discrete period, which they designated the "window of infectivity" [1]. However, we have observed clinically that very severe caries can occur before this discrete period of MS infectivity in infants and toddlers, posing a question regarding the etiology of caries in these children. Furthermore, interesting results have been reported by many previous studies. MS can be detected in some caries-free children, while some subjects with severe caries do not have detectable levels of *S. mutans* [7]. Therefore, MS may not be the only cariogenic bacteria; other taxa may also play a role. Thus, the pathogenesis of dental caries is not entirely clear, and use of new methods is necessary to identify the unknown potential pathogens.

Many studies of oral microbial diversity have been conducted. Radford *et al*. [8] examined 1393 1-year-old infants and took saliva samples (using the tongue-loop method) for microbiological culture. Infants with caries had higher isolation frequencies and higher counts of *S. mutans*, lactobacilli and yeasts (but not *S. sobrinus*) compared with those who were clinically caries-free. Li *et al.* [9] evaluated the difference in oral microbial diversity between 2 to 8-year-old children with severe early childhood caries (S-ECC) and caries-free controls by means of a cultivation-independent approach called denaturing gradient gel electrophoresis (DGGE). They observed that the mean species richness of the bacterial population was significantly greater in the caries-free children than in the S-ECC children, suggesting that the microbial diversity and complexity of the microbial biota in dental plaque are significantly lower in S-ECC children than in caries-free children. Aas *et al.* [5] used *16S rRNA* gene sequencing and a reverse-capture checkerboard assay to detect all bacterial species associated with caries in primary and permanent teeth in subjects ranging from 2 to 21 years old. They found that 10 to 20% of subjects with severe caries may not have detectable levels of *S. mutans* but do have other acid-producing species. Furthermore, in some carious lesions, *S. mutans* may be a minor bacterial component of dental plaque. Species in addition to *S. mutans*; *e.g.*, *Veillonella*, *Lactobacillus*, *Bifidobacterium*, *Propionibacterium*, low-pH non-*S.mutans* streptococci, *Actinomyces*, and *Atopobium*, may also play an important role in caries production. Additionally, *Actinomyces* spp. and non-*S. mutans* streptococci may be involved in the initiation of the disease.

Considerable differences in bacterial composition and diversity between individual sites and surfaces of the oral cavity have been demonstrated [10]. Given that the tooth surfaces are the sites where dental caries take place, the use of saliva as a proxy for bacterial composition at those sites may not provide meaningful correlations between bacterial composition and disease status in epidemiological and etiological studies. In addition, some studies have found an association between microbiota and disease in plaque samples but not saliva, in both gingivitis and dental caries [11], [12]. Although dental plaque is recognized as a complex microbial system, there are substantially fewer experimental studies which have investigated dental plaque from a microbial ecology perspective than those that have described a single species or selected bacterial consortia. Therefore, exploration of the oral microbiota from a microbial ecology perspective during caries causation and development is key for a more complete understanding of the etiology of dental caries. [13].

The earliest studies used culture methods, but many bacteria were unable to be cultured. In contrast to conventional culture methods, molecular techniques have the advantage of detecting difficult-to-grow bacteria. However, only expected species have been investigated with any frequency because the number of target bacteria for polymerase chain reaction (PCR) techniques or checkerboard DNA–DNA hybridization assay is restricted. [14] Human Oral Microbe Identification Microarray (HOMIM) and *16S rRNA* gene pyrosequencing are two common high-throughput oral microbiome assays that enable microbiome assessment beyond the capacity of RFLPs [15].

*16S rRNA* gene pyrosequencing is a broad-based sequencing approach, using PCR primers on highly conserved regions to amplify of a segment of the *16S rRNA* gene, followed by DNA pyrosequencing to identify unique sequence reads. Compared to traditional sequencing techniques, such as Sanger sequencing, pyrosequencing provides a larger number of readings and greater depth of coverage in a cost-efficient manner [16].

The *16S rRNA* gene pyrosequencing method has been used widely in the study of oral diseases and systemic diseases, including caries [17], periodontitis [18], [19], oral squamous cell carcinoma [20] and gastrointestinal cancer [16]. However, detection of the oral microbes that contribute to severe early childhood caries using this method remains problematic.

## Materials and Methods

### Ethics Statement

Written informed consent was obtained from the parents of all children in this study. The study design, protocol, and informed consent were approved by Ethics Committee of Peking University Health Science Center (IRB00001052-5132).

### Selection of study subjects

Ten children less than 30 months of age with dental caries were recruited from the dental clinics at the Departments of Pediatric Dentistry at Peking University’s School of Stomatology. Inclusion criteria were that children were medically healthy, had primary dentition without eruption of second primary molars, had at least two caries in both anterior and posterior teeth, had no enamel or dentin hypoplasia detectable visually, had not used antibiotics within the preceding 2 weeks, and had not received any treatment or fluoride treatment of dental caries. Consecutive children who fulfilled the inclusion criteria were enrolled.

Nine caries-free children without eruption of the second primary molars and who had no caries (including white-spot) lesions or restorations formed the control group. They were selected from childcare facilities and children who presented for oral examination.

### Clinical examination, collection of plaque

The children were examined in the dental clinics at the Dental Clinic and Pediatric Dentistry Department at the School of Stomatology of Peking University. The dental examinations were performed by the same physician, through visual/tactile methods, so as to determine the incidence of caries, based on the number of decayed teeth (dt) score (since there is no missing and filled teeth in these children). The teeth were gently dried with a piece of cotton, and a dental mirror was used to detect cavities and enamel hypoplasia. An assistant helped to record the findings, and the dental examination was usually completed within 15 min. Caries were charted using criteria of the WHO criteria (1987).

For each child, dental plaque was sampled from intact enamel. The samples were collected by means of a sterile excavating-spoon hand-instrument and were placed immediately in an Eppendorf tube containing 1 ml of sodium thiosulfate solution.

### Laboratory methods

#### (1) Extraction of chromosomal DNA

The plaque was washed twice with TE buffer (10 mM Tris-HCl, 1 M EDTA, pH 8.0). Bacterial DNA was isolated and purified using a Wizard Genomic DNA Purification Kit, according to the manufacturer’s instructions (Promega, Madison, USA). The final quantity and quality of the DNA was evaluated using a DU-7400 UV–VIS spectrophotometer at OD260/OD280. A standard concentration of 10 ng/µl was prepared for each individual sample for all PCR assays.

#### (2) Pyrosequencing

Bacterial *16S rRNA* gene amplification, cloning, and sequencing of the polymerase chain reaction (PCR) products were performed at the laboratory of BGI (Huada Gene Institute).

PCR amplification of the V1-V3 region of bacterial *16S rRNA* gene was performed using universal primers (27F 5′-AGAGTTTGATCCTGGCTCAG-3′, 534R 5’′-TTACCGCGGCTGCTGGCAC-3′) incorporating the FLX Titanium adapters and a sample barcode sequences. The PCR condition is as follows: 2 min initial denaturation at 95°C; 30 cycles of denaturation at 95°C (20 s), annealing at 56°C (30 s), elongation at 72°C (45 s); and final extension at 72°C for 7 min. The PCR products were separated by 1% agarose gel electrophoresis and the about 500bp fragment were purified by using the QIA quick Gel extraction kit (Qiagen). Equal concentrations of amplicons were pooled from each sample. Emulsion PCR and sequencing were performed according to the manufacturer’s recommendations. [21].

#### (3) Bioinformatic Analysis

The multiplexed samples were deconvoluted computationally using customized Perl scripts, based on the presence of the unique barcodes assigned to each sample. The barcodes and primers were then trimmed off and the low quality sequences were removed. The high quality sequence reads were treated with the mothur v.1.27.0 Standard Operation Procedure (SOP). [22] The community structure of a sample was calculated based on the membership and relative abundance, based on proportion of reads, of taxonomic groups in the sample.

#### (4) Statistical Analysis

The UniFrac distance [23], [24] metrics analysis was performed based on OTUs phylogenetic tree and abundance in each sample, and principal coordinate analysis (PCoA) was conducted according to the matrix of UniFrac distance. UniFrac distance, rarefaction and alpha-diversity were calculated by Mothur (v1.27). To select OTUs that exhibited significance in the structural segregation between groups, a Paired Sample T test was performed based on the OTUs abundance, Paired Sample T test and PCA was performed by R statistical software (2.15.2).

## Results and Discussion

With the advent of molecular techniques, bacterial diversity and community structure in different microhabitats have been investigated using molecular fingerprinting methods such as PCR-DGGE and sequence analysis of microbial *16S rRNA* genes and other universal targets (such as cpn60). [25], [26] However, it should be noted that based on the PCR-DGGE profiles, only predominant members of the bacterial community can be represented. The *16S rRNA* pyrosequencing assay is designed to detect broadranged microbiome profiles, particularly in rarer taxa, while the custom-designed HOMIM is developed to specifically capture the major oral microbiome species, which are covered by the reference sequences. Improvements in pyrosequencing enable a dramatic increase in throughput via parallel in-depth analysis of large scale samples with limited sample processing and lower costs. [27].

### Subjects

Plaque samples were collected from 10 children with caries who were at a mean age of 19.1±3.5 months, and nine caries-free children with a mean age of 19.3±3.2 months old. Chi-square analysis indicated no significant differences between the groups in term of gender, and the mean age difference between the two groups was not statistically significant by *t*-test. In the caries group, 8 out of these 10 children had 16 teeth erupted, while the other 2 caries children respectively had 15 and 12 teeth erupted. Furthermore, 6 out of the 9 caries-free children had 16 teeth erupted, while the other 3 children respectively had 13, 10 and 15 teeth erupted. No significant difference was found between the two groups. The mean decayed teeth number of the caries children is 6.9. (Table 1).

**Table 1:** Age, sex information of children in study and number of sequences obtained.

Sample	Sex	Age (month)	Teeth number	dt	Raw reads num	Final reads num	OTU num*
**C1**	female	16.5	16	8	25656	5491	162
**C2**	male	19	16	6	20577	3135	139
**C3**	female	17.5	16	6	20733	2117	125
**C4**	female	17.5	16	12	24079	3924	143
**C5**	male	24.5	16	6	24224	5638	161
**C6**	male	19.5	16	4	30617	4221	219
**C7**	female	17.5	16	5	25274	4613	189
**C8**	female	24	15	4	24980	4979	160
**C9**	male	22	16	8	21847	4863	255
**C10**	female	13	12	10	23635	3108	142
**CF1**	male	18.5	16	0	23782	5644	229
**CF2**	male	16	16	0	26730	7990	249
**CF3**	female	13.5	16	0	25073	5728	236
**CF4**	female	18	16	0	20226	4351	248
**CF5**	male	19	13	0	27058	2556	156
**CF6**	female	18	10	0	19982	3120	209
**CF7**	female	22	15	0	17482	3151	149
**CF8**	male	22	16	0	18853	3560	182
**CF9**	male	24	16	0	8762	1938	149

^*^ Operational Taxonomical Unit (OTU) at 3% dissimilarity based upon high quality sequence selected MOTHUR observed rarefaction.

According to the inclusion criteria, all these 10 caries children suffered from very severe caries at a very young age. In the light of literature search, subjects included in previous researches on oral microbiota of severe early childhood caries were mostly 3–6 years of age, when all primary teeth have fully erupted [12], [28], or even wider ranges of ages [5], [9], [29]. However, our study focused on children only less than 25 months old with second primary molars unerrupted. Their ages were relatively small and concentrated, and the deciduous dentition was not fully established at this time. In addition, the inclusion criteria of the caries group in some previous researches was only “with caries” [5], [30], while we chose the caries group children by at least two caries in both anterior and posterior teeth, which meant they had at least four decayed teeth in no more than sixteen primary teeth.

This suggested that these children had a much more severe caries condition and could be considered as populations who were extremely susceptible to caries decay. Research about oral microflora in these populations gave us an insight into the constitution of microorganisms in such severe caries condition, and might help us better understand cariogenetic bacteria at such a young age. Besides, analysis of microbial diversity in this particular period may give us more information on the caries etiology and more instructions about treatment and prevention of this disease [31]. According to literature search, there is no expatiation of supragingival plaque bacterial diversity using sequencing method at such young age and with such severe caries. Moreover, there was no particular description and comparative studies about plaque microbiota on intact enamel of such little children, caries and caries-free alike. [30]

### OTU diversity in the plaque samples

**Figure 1:**
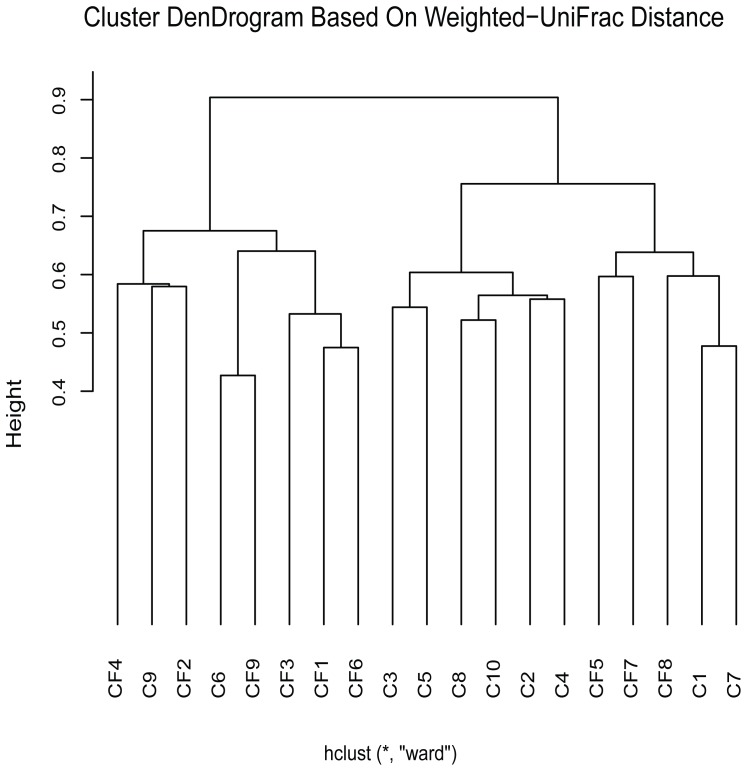
Weighted Unifrac clustering results of the subjects.

We generated an average of over 24,000 sequences from samples from children with caries and over 20,000 sequences from samples from caries-free children, which were then analyzed (Table 1). Operational taxonomical unit (OTU) diversity was assessed using Mothur [22], as this method is well documented for evaluation of alpha diversity. Mothur identified a lower number of OTUs for the children with caries (mean OTU at 3% dissimilarity = 170) than those caries-free (mean OTU at 3% dissimilarity = 201), but found no significant difference. The alpha diversity analysis is presented in Table S1. The dental plaque communities were analyzed at a 3% dissimilarity level, the richness of the total amount of bacteria in plaque was estimated by ACE and Chao. The diversity of the plaque microbiota was estimated by Shannon and Simpson. Comparisons of the diversity index demonstrated that there was no significant difference between these severe caries and caries-free children at 97% identity. Based on the species-profiling table, we assessed the content of each sample group and calculated the beta diversity of the OTU level between the samples. Hierarchical clustering was performed using weighted Unifrac index (Fig. 1).Within these subgroups, only one subgroup contained only caries plaque samples; the other subgroups comprised plaque samples from both groups. Based on this result, we speculate that the mechanisms underlying tooth decay cannot be explained by only the abundance of bacteria OTUs in plaque. Thus, we further compared the bacterial composition of the caries and caries-free groups.

### Composition of the plaque microbiome

To assess taxonomic diversity, each trimmed reading (tag sequence) served as a query to identify its closest match in a reference database. The ensemble of sequences in this study provided a broad view of the plaque microbiota. A total of 8 phyla, 15 classes, 21 orders, 30 families, 41 genera and 99 species were represented in these plaque samples.

**Figure 2:**
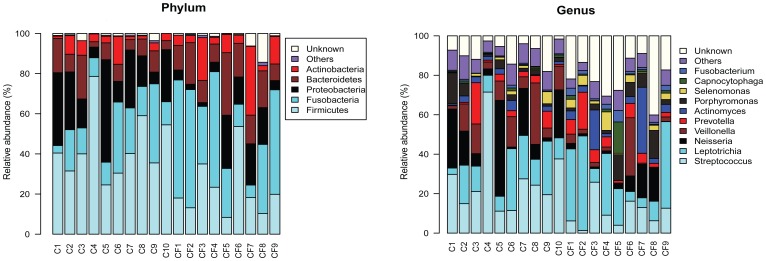
(a) Abundance and prevalence of the different bacterial phyla in 19 plaque samples (b) Abundance and prevalence of the different bacterial genera in 19 plaque samples.

Eight phyla were represented in the plaque samples (Fig. 2a).The most abundant were *Firmicutes* (around 25–79% of total sequences in caries group and 8–53% of total sequences in caries-free group), *Fusobacteria* (around 4–39% of total sequences in caries group and 6–59% of total sequences in caries-free group), *Proteobacteria* (around 5–51% of total sequences in caries group and 3–26% of total sequences in caries-free group), *Bacteroidetes* (around 3–22% of total sequences in caries group and 8–31% of total sequences in caries-free group) and *Actinobacteria* (around 1–14% of total sequences in caries group and 3–21% of total sequences in caries-free group). These five predominant phyla constituted 97.48% of the total microbiota. But no one phylum among the five above was significantly different between caries-active and caries-free samples (p>0.05). The phylum of *Spirochaetes* was only found in caries-free samples with low relative abundance (around 0–0.09% of total sequences) in the total sequences. The remaining bacteria belonged to the candidate division TM7 or SR1 (around 0–1.69% of total sequences).

At the genus level, a total of 41 genera were represented, while 38 genera found in caries samples, 37 genera found in caries-free samples. (Fig. 2b) Among these genera, 34 genera were shared by both groups. Seven genera (*Leptotrichia, Streptococcus, Actinomyces, Prevotella, Porphyromonas Neisseria, and Veillonella*) constituted roughly 80% of the plaque microbiota of the caries samples and 64% of the plaque microbiota of the caries-free samples. A total of 20 genera (including the seven genera mentioned above plus *Kingella, Abiotrophia, Granulicatella, Selenomonas, Rothia, Corynebacterium, Capnocytophaga, Fusobacterium, Gemella, TM7 genus incertae sedis, Campylobacter, Eikenella, Ottowia*) constituted roughly 91% of the plaque microbiota of the caries samples and 78% of the plaque microbiota of the caries-free samples. A complete list of bacterial phylotypes per sample is provided in Table S2.

In these young children without the second primary molars erupted, the genera composition type were roughly the same between these caries and caries-free children, while their contents were quite different. The seven major genera constituted over 60% of the total microbiota in both groups. *Streptococcus, Neisseria* and *Veillonella* presented higher contents in the caries group than in the caries-free group, while *Leptotrichia, Actinomyces, Prevotella* and *Porphyromonas* presented the opposite trend. But the statistics of these five genera showed no significant difference between caries and caries-free samples (P>0.05). The genera which were detected only in the caries group were *Lactobacillus, Acinetobacter, Anaeroglobus, Schlegelella,* the genera which were detected only in the caries-free group were *Treponema, Johnsonella* and *Dechloromonas*. All these six genera were found with low relative abundance (around 0.002–0.019%) in the total sequences.

Our study represented supragingival plaque bacterial diversity n primary dentition with second primary molars unerrupted for the first time. According to previous studies which focused on children aged 3–6 years old with and without dental caries by Ling *et.al* [12], the total bacteria phylum types and the predominant phyla types in supragingival plaque samples displayed quite the same as our results. However, 14 phyla were found in the study of Jiang *et.al* [28], in which the five predominant phyla were the same as in our study, with other phyla presented in relatively low proportions.

At the genus level, in Ling’s study [12], about 126 other genera (153 in total) were found in dental plaque of 3-6-year old children, with 13 genera (*Actinomyces, Capnocytophaga, Corynebacterium, Fusobacterium, Haemophilus, Granulicatella, Kingella, Leptotrichia, Neisseria, Prevotella, Streptococcus, Thiomonas,* and *Veillonell*a) constituted roughly 80% of the oral microbiota. While in Jiang’s study [28], the sequences from the plaque samples contained 63 different genera, with the majority of the sequences belonged to 10 genera: *Leptotrichia, Neisseria, Streptococcus, Corynebacterium, Derxia, Prevotella, Capnocytophaga, Veillonella, Fusobacterium,* and *Porphyromonas*, which constituted 81% of the total oral microbiota.

### Taxonomic analysis and comparison of the host populations

The microbiotas of plaque from the caries and caries-free groups were also compared. Among all taxa, we were able to pinpoint several “caries-associated” (differentially distributed in caries microbiomes yet present in both populations; including the two circumstances of ‘caries-enriched’ and ‘caries-depleted’) taxa at each of those six taxonomy levels. The caries-enriched taxa included *Firmicutes* and *Proteobacteria* at Phylum, *Bacilli* at Class, *Lactobacillales* at Order, *Streptococcacea* at Family, and *Streptococcus mutans* at Species.

**Figure 3:**
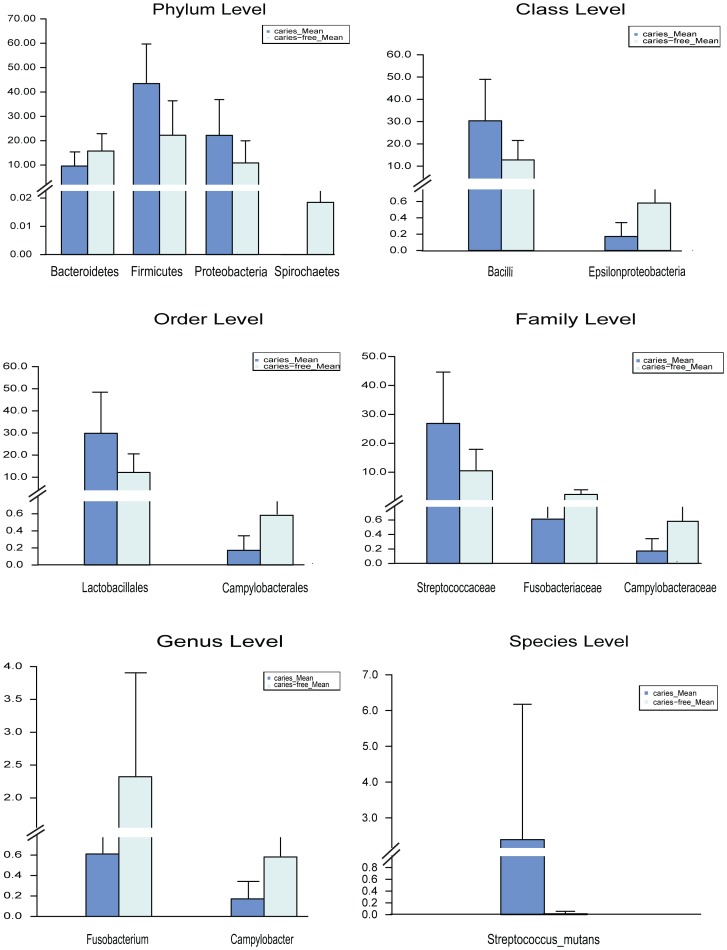
Mean levels of the bacteria which presented significantly different contents in caries and caries-free group. From a to f shows the different taxonomic levels of bacteria (a: phylum level; b: class level; c: order level; d: family level; e: genus level; f: species level).

In contrast, the caries-depleted taxa included Bacteroidetes and Spirochaetes at Phylum, Epsilonproteobacteria at Class, Campylobacterales at Order, Campylobacteraceae and Fusobacteriaceae at Family, Campylobacter and Fusobacterium at Genus, none at Species (Figure 3).

In Ling’s study, six genera (*Streptococcus, Veillonella, Actinomyces, Granulicatella, Leptotrichia,* and *Thiomonas*), which constituted a large proportion of oral microbiota, were significantly different between caries-active and caries-free samples in plaque of 3-6 year old children (p<0.05). [12] In Jiang’ study, three genera (*Streptococcus, Granulicatella,* and *Actinomyces*) exhibited a relatively increased abundance in severe ECC subjects, whereas caries-free subjects exhibited a relatively increased abundance of *Aestuariimicrobium* (P<0.05) [28]. However, only two caries-depleted genera (*Campylobacter* and *Fusobacterium*) were found significantly distributed in children aged 16-25 months old in our study (P<0.05). Some undisputed cariogenic genera, such as *Streptococcus,* didn’t show significantly different contents between the two groups. Except for the reasons of sample size, it still needs further investigation on the species level of these genera.

A list of selected bacterial phylotypes that were overrepresented in the caries group is shown in Table 2. Further work should elucidate the potential role these bacteria play in the progression of caries, as well as their synergistic and antagonistic interactions.

**Table 2:** Selected bacterial phylotypes identified in caries subjects and their putative virulence properties.

Bacterial phylotypes	Characteristics	References
*Streptococcus mutans*	Cariogenic, principle acid producer	Boue *et al.* (1987) [36]
*Streptococcus sobrinus*	Cariogenic, acid producer	Kohler *et al.* (1995) [37]
*Granulicatella adiacens*	Causes infections	Hepburn *et al.* (2003) [38]
*Leptotrichia hongkongensis*	Natural reservoir in oral cavity	Woo *et al.* (2010) [39]
*Prevotella histicola*	Exists both in oral squamous cell carcinoma tissue and non-tumorous mucosal tissue	Downes *et al.* (2008) [32]

**Figure 4:**
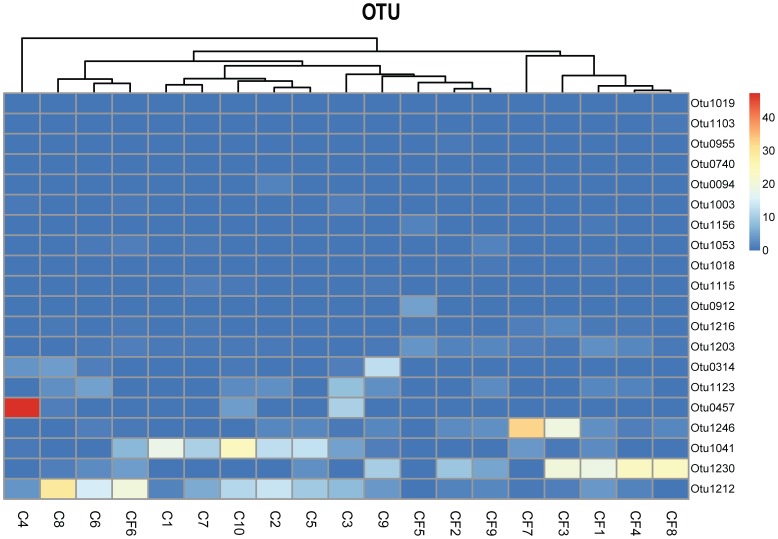
Relative abundance of OTUs in the samples from the two groups. Colors reflect relative abundance from low (blue) to high (red). Sample CF6 (caries-free sample) clusters together with the caries samples.

The heatmap (Figure. 4) of the 20 most predominant OTUs in caries and caries-free groups showed that the caries-free children (except one) clustered apart from caries children. Sample CF6 (a caries-free sample) clustered together with the caries samples. This child still needs further inspection by longitudinal studies. In the heatmap, OTU1212(*Veillonella*), OTU1041(*Neisseria*), OTU0457(*Streptococcus*) were found at obviously higher contents in the caries samples, indicating these three OTUs were very important factors in determining the caries state. Especially for sample C4, only OTU0457 presented a high content while the remaining major OTUs were all at very low concentrations. On the contrary, OTU1230(*Fusobacteriales*) and OTU1246(*Actinomyces*) were found obviously at higher contents in the caries-free samples, indicating these two OTUs were very important factors in determining the caries-free state.

#### Principal Component Analysis (PCA)

**Figure 5:**
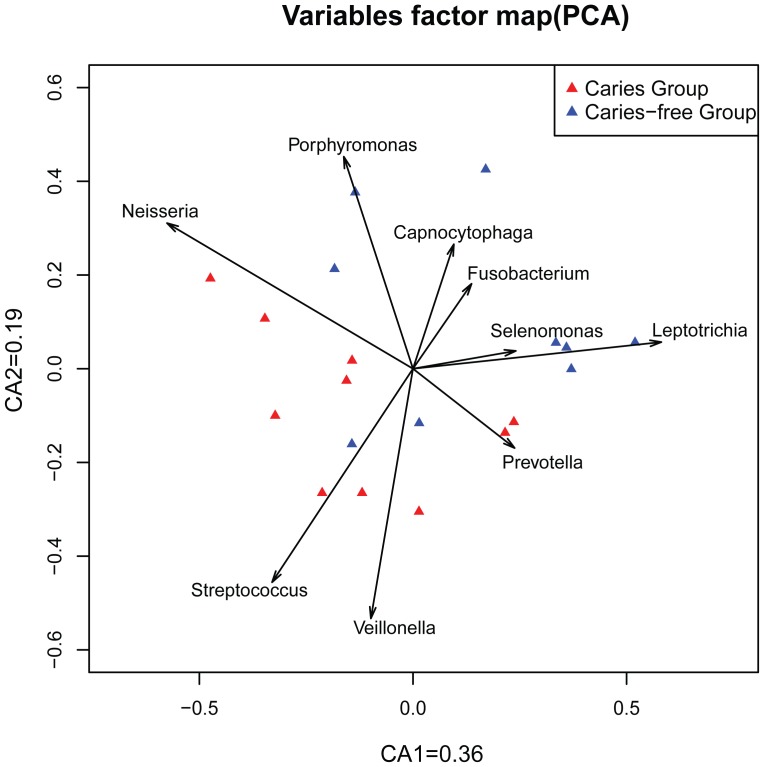
Principal Component Analysis of the genus information for caries and caries-free groups.

To assess the classification of the bacteria into these two groups, Principal Component Analysis (PCA) was implemented. (Figure. 5) Based on genus information, the caries-free samples appeared to cluster together, in the opposite direction of the caries samples, with two samples mixed into the caries cluster.

Figure 5 also showed genera that closely related to these children’s caries state. The caries-related genera included *Streptococcus* and *Veillonella*; while *Leptotrichia*, *Selenomonas*, *Fusobacterium*, *Capnocytophaga* and *Porphyromonas* were more related to the caries-free samples. These genera likely played a deciduous role in dividing the groups. *Neisseria* and *Prevotella* presented approximately in between.

*Streptococcus* at *Genus* was detected in all children (Fig. 2b), but represented a higher proportion of total flora in caries group than in the caries-free group. This was also reflected in the PCA analysis, suggesting the difference in the *Streptococcus* proportion of the total flora to be one of the main reasons for the distinction between the plaque floras of the two groups.

Although previous studies reported that *Bacteroidetes* constituted a significantly higher proportion of caries supragingival samples than in caries-free samples [30], we obtained some interesting findings (Table S2). A special OTU (OTU0491), which belonged to *Bacteroidetes*, was found in none of the caries samples and was barely detectable in the caries-free group (mean = 0.0171). Another special OTU0955 (*Prevotella*, which also belonged to *Bacteroidetes*, mean (C)  = 0.0041, mean (CF) = 0.1348) was present at a much higher level in the caries-free group than the caries group. In addition, several species of *Prevotella* were present in varying amounts between the caries and caries-free groups. (This was consistent with the middle position of *Prevotella* in the PCA figure.) OTU1216 (*Prevotella loescheii*, mean (C)  = 0.2580, mean (CF) = 0.8347), OTU0740 (*Prevotella maculosa*, mean (C)  = 0.0186, mean (CF) = 0.1259), OTU0659 (*Prevotella micans*, mean (C)  = 0.0113, mean (CF) = 0.0865), OTU1019 (*Prevotella saccharolytica*, mean (C)  = 0.0092, mean (CF) = 0.1216) were as prevalent as OTU0955. However, the OTU1003 (*Prevotella histicola*, mean (C)  = 0.2461, mean (CF)  = 0.0641) exhibited the opposite trend, being found at a markedly higher incidence in the caries group.

*Prevotella histicola* was isolated from human oral mucosal tissue in 2008 [32] and could exist in both oral squamous cell carcinoma tissue and non-tumorous mucosal tissue [33]. These findings further supported the ecological plaque hypothesis that caries result from a shift in the balance of the resident microflora driven by changes in local environmental conditions [34]. The proportion of certain species or strains with similar biological properties may decrease, others may increase. We hypothesize that the increased bacteria taxa, such as *Prevotella histicola*, exhibit characteristics that facilitate their adaptation to the new environment. These phenomena require further investigation.

### Novel oral bacterial phylotypes

Of the bacterial phylotypes obtained (excluding the unclassified), 7 strains were not listed in neither the Human Oral Microbiome Database (HOMD) (http://www.homd.org) nor http://microbiome.osu.edu (an oral reference database previously published in PLoS One), including *Actinomyces timonensis, Eubacterium saburreum, Selenomonas bovis, Streptobacillus moniliformis, Ottowia thiooxydans, Schlegelella thermodepolymerans* and *Dechloromonas agitate.* (Table 3) Of these, *Selenomonas bovis* was detected in only two caries samples. However, it didn’t present at significant different levels between the groups. Further study of these bacteria may lead to fresh understanding of the oral microbiome and severe childhood caries.

**Table 3:** Species detected in plaque samples that were not listed in HOMD nor http://microbiome.osu.edu.

Species	Existence	References
*Actinomyces timonensis*	Human clinical osteo-articular sample	Renvoise et al. (2010) [40]
*Eubacterium saburreum*	Periodontal pocket	Reynaud et al. (2001) [41]
*Selenomonas bovis*	Yak rumen contents	Zhang et al. (2009) [42]
*Streptobacillus moniliformis*	Pus and blood	Hagelskjaer et al. (1998) [43]
*Ottowia thiooxydans*	Activated sludge	Spring et al. (2004) [44]
*Schlegelella thermodepolymerans*	Compost	Romen et al. (2004) [45]
*Dechloromonas agitata*	Environment	Achenbach et al. (2001) [46]

To assess the level of microbial conservation among these children, we evaluated the existence of a ‘core’ plaque microbiome shared among the subjects. The degrees of OTU sharing and unsharing were determined, respectively, for both caries and caries-free children. After removing the duplicate sequences, the caries and caries-free groups presented 1280 OTUs with 351 shared OTUs, and 428 and 501 OTUs were unique to the respective groups. (Figue. 6) Comparison within the groups demonstrated nine (1.16% in the caries group and 1.06% in the caries-free group) OTUs in all samples, while no specific OTU was detected to be present in all the samples in one group but none in the other group. Therefore, in both populations, the degree of shared organism lineages (as defined by species-level OTUs) among individual supragingival plaque microbiomes was minimal.

**Figure 6:**
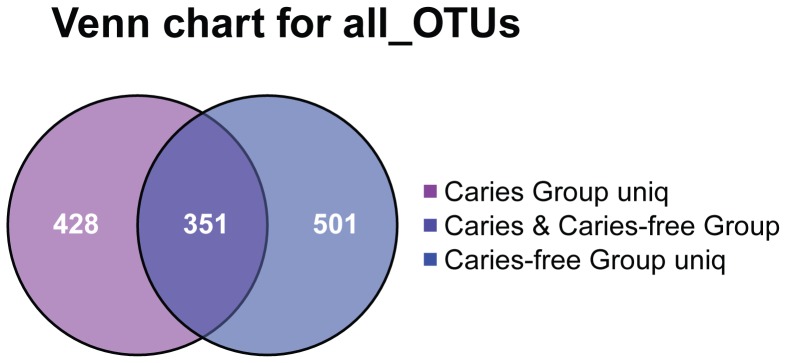
Venn diagram of the number of OTUs common/unique within caries and caries-free group. The interior of each circle symbolically represents the number of observed OTUs in the certain sample/group. The overlapping area or intersection would represent the set of OTU commonly present in the counterpart samples/groups. Likewise, the single-layer zone represents the number of OTUs uniquely found in the certain sample/group.

In 2009, Zaura *et. al* [26] investigated the diversity and uniqueness of individual oral microbiomes at a resolution of GS-FLX sequencing. Their results showed that saliva microbiomes from three healthy Caucasian male adults shared 387 (47%) of 818 total OTUs, supporting the concept of a core microbiome at health. However, later research by Yang *et al.* [35] supported the absence of a species-level organismal ‘core’ of saliva microbiome among adult human hosts based on the observation that a gradual decrease of OTU sharing with every individual addition of hosts. Our data indicated there was a minimal ‘core’ of plaque microbiomes in these children populations, caries or caries-free hosts alike, which much resembled the Yang research.

## Conclusions

Comprehensive investigation of the composition of the oral microbial ecosystem is essential for a better understanding of the etiology of dental caries. Although there are several previous studies that focus on the oral microbiota of supragingival plaque of children with and without dental caries [12], our study represented for the first time using pyrosequencing to elucidate and monitor supragingival plaque bacterial diversity at such a young age with second primary molars unerrupted. 41 genera belonging to eight phyla were represented in the plaque samples. Our results showed that five predominant phyla (*Firmicute, Fusobacteria, Proteobacteria, Bacteroidetes* and *Actinobacteria*) and seven genera (*Leptotrichia, Streptococcus, Actinomyces, Prevotella, Porphyromonas, Neisseria, and Veillonella*) constituted a majority of contents of the total microbiota, independent of the presence or absence of caries. However, distinctions were revealed between caries and caries-free microbiomes in terms of microbial community structure. We were able to pintpoint several “caries-associated” taxa between the two populations. Principal Component Analysis (PCA) presented that caries-related genera included *Streptococcus* and *Veillonella*; while *Leptotrichia*, *Selenomonas*, *Fusobacterium*, *Capnocytophaga* and *Porphyromonas* were more related to the caries-free samples. *Neisseria* and *Prevotella* presented approximately in between. In both groups, the degree of shared organism lineages (as defined by species-level OTUs) among individual supragingival plaque microbiomes was minimal. Our approach and findings could be extended to correlating microbiomic changes after occlusion establishment and caries treatment.

## Supporting Information

Table S1 **Alpha diversity analysis of all the samples at 3% dissimilarity.**
(DOC)

Table S2 **Complete list of bacterial phylotypes.**
(XLS)
