# Supplementary material for: Plaque Bacterial Microbiome Diversity in Children Younger than 30 Months with or without Caries Prior to Eruption of Second Primary Molars
Source: PLoS One. 2014 Feb 28;9(2):e89269. doi: 10.1371/journal.pone.0089269 (PMC3938432; doi:10.1371/journal.pone.0089269)
Supplement: Table S1 — Alpha diversity analysis of all the samples at 3% dissimilarity. (DOC) [file pone.0089269.s001.doc]

**Table S1.** Alpha diversity analysis of all the samples at 3% dissimilarity

| **Sample ID** | **chao** | **ace** | **jackknife** | **shannon** | **npshannon** | **simpson** |
| --- | --- | --- | --- | --- | --- | --- |
| **C1** | 288.56 | 334.05 | 322.16 | 2.89 | 2.94 | 0.110 |
| **C2** | 201.64 | 249.01 | 204.12 | 3.36 | 3.42 | 0.061 |
| **C3** | 198.50 | 262.78 | 216.55 | 3.55 | 3.62 | 0.048 |
| **C4** | 225.22 | 289.70 | 243.18 | 2.47 | 2.54 | 0.243 |
| **C5** | 261.50 | 345.28 | 289.20 | 2.85 | 2.90 | 0.144 |
| **C6** | 339.68 | 472.58 | 381.86 | 3.57 | 3.64 | 0.076 |
| **C7** | 468.07 | 503.71 | 641.83 | 3.23 | 3.30 | 0.078 |
| **C8** | 430.10 | 433.25 | 458.86 | 3.00 | 3.05 | 0.123 |
| **C9** | 443.36 | 612.70 | 550.41 | 3.80 | 3.88 | 0.048 |
| **C10** | 272.00 | 338.65 | 312.05 | 3.11 | 3.18 | 0.094 |
| **CF1** | 392.80 | 437.15 | 4678.12 | 3.18 | 3.24 | 0.120 |
| **CF2** | 378.64 | 426.54 | 410.85 | 2.92 | 2.97 | 0.131 |
| **CF3** | 374.00 | 439.80 | 497.13 | 3.13 | 3.19 | 0.118 |
| **CF4** | 519.44 | 675.68 | 873.51 | 3.49 | 3.58 | 0.087 |
| **CF5** | 237.38 | 301.62 | 282.03 | 3.46 | 3.55 | 0.058 |
| **CF6** | 407.12 | 561.73 | 451.81 | 3.61 | 3.71 | 0.064 |
| **CF7** | 248.04 | 364.93 | 270.90 | 2.97 | 3.05 | 0.135 |
| **CF8** | 285.12 | 303.58 | 322.50 | 3.05 | 3.14 | 0.107 |
| **CF9** | 310.00 | 344.23 | 340.99 | 3.29 | 3.39 | 0.113 |
